# Supplementary material for: Causal Relationship between Aging and Anorexia Nervosa: A White-Matter-Microstructure-Mediated Mendelian Randomization Analysis
Source: Biomedicines. 2024 Aug 16;12(8):1874. doi: 10.3390/biomedicines12081874 (PMC11351342; doi:10.3390/biomedicines12081874)
Supplement: Supplementary file 1 [file biomedicines-12-01874-s001.zip › Supplementary Figure/Figure S4/Singe SNP plot of MR analysis for TL to ubm-b-1584.pdf]

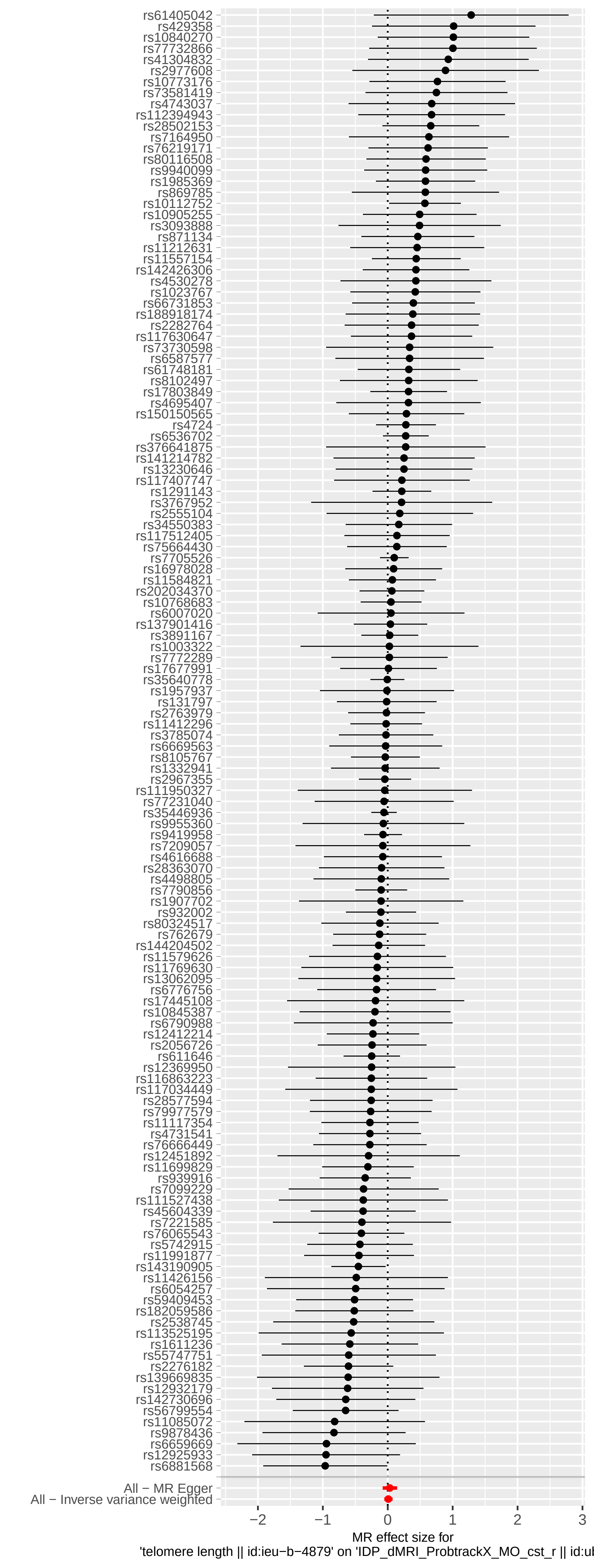
rs61405042
rs429358
rs10840270
rs77732866
rs41304832
rs2977608
rs10773176
rs73581419
rs4743037
rs112394943
rs28502153
rs7164950
rs76219171
rs80116508
rs9940099
rs1985369
rs869785
rs10112752
rs10905255
rs3093888
rs871134
rs11212631
rs11557154
rs142426306
rs4530278
rs1023767
rs66731853
rs188918174
rs2282764
rs117630647
rs73730598
rs6587577
rs61748181
rs8102497
rs17803849
rs4695407
rs150150565
rs4724
rs6536702
rs376641875
rs141214782
rs13230646
rs117407747
rs1291143
rs3767952
rs2555104
rs34550383
rs117512405
rs75664430
rs7705526
rs16978028
rs11584821
rs202034370
rs10768683
rs6007020
rs137901416
rs3891167
rs1003322
rs7772289
rs17677991
rs35640778
rs1957937
rs131797
rs2763979
rs11412296
rs3785074
rs6669563
rs8105767
rs1332941
rs2967355
rs111950327
rs77231040
rs35446936
rs9955360
rs9419958
rs7209057
rs4616688
rs28363070
rs4498805
rs7790856
rs1907702
rs932002
rs80324517
rs762679
rs144204502
rs11579626
rs11769630
rs13062095
rs6776756
rs17445108
rs10845387
rs6790988
rs12412214
rs2056726
rs611646
rs12369950
rs116863223
rs117034449
rs28577594
rs79977579
rs11117354
rs4731541
rs76666449
rs12451892
rs11699829
rs939916
rs7099229
rs111527438
rs45604339
rs7221585
rs76065543
rs5742915
rs11991877
rs143190905
rs11426156
rs6054257
rs59409453
rs182059586
rs2538745
rs113525195
rs1611236
rs55747751
rs2276182
rs139669835
rs12932179
rs142730696
rs56799554
rs11085072
rs9878436
rs6659669
rs12925933
rs6881568
All − MR Egger
All − Inverse variance weighted
−2
−1
0
1
2
3
MR effect size for
'telomere length || id:ieu−b−4879' on 'IDP_dMRI_ProbtrackX_MO_cst_r || id:ul
